# Exosomes from differentially activated macrophages influence dormancy or resurgence of breast cancer cells within bone marrow stroma

**DOI:** 10.1038/s41419-019-1304-z

**Published:** 2019-01-25

**Authors:** Nykia D. Walker, Michael Elias, Khadidiatou Guiro, Ranvir Bhatia, Steven J. Greco, Margarette Bryan, Marina Gergues, Oleta A. Sandiford, Nicholas M. Ponzio, Samuel J. Leibovich, Pranela Rameshwar

**Affiliations:** 1 Rutgers New Jersey Medical School (NJMS), Newark, NJ USA; 2 Rutgers Graduate School of Biomedical Sciences, Newark, NJ USA; 3 Dept of Pathology and Laboratory Medicine, Philadelphia, PA USA; 4 NJMS, Department of Cell Biology and Molecular Medicine, Newark, NJ USA

## Abstract

Breast cancer (BC) cells (BCCs) can retain cellular quiescence for decades, a phenomenon referred to as dormancy. BCCs show preference for the bone marrow (BM) where they can remain dormant for decades. Targeting BCCs within the BM is a challenge since the dormant BCCs reside within BM stroma, also residence for hematopoietic stem cells (HSCs). Dormant BCCs could behave as cancer stem cells (CSCs). The CSCs and HSCs are similar by function and also, by commonly expressed genes. The method by which dormant BCCs transition into clinically metastatic cells remains unclear. This study tested the hypothesis that macrophages (MΦs) within BM stroma, facilitates dormancy or reverse this state into metastatic cells. MΦs exhibiting an M2 phenotype constitute ~10% of cultured BM stroma. The M2 MΦs form gap junctional intercellular communication (GJIC) with CSCs, resulting in cycling quiescence, reduced proliferation and carboplatin resistance. In contrast, MΦs expressing the M1 phenotype reversed BC dormancy. Activation of M2a MΦs via the toll-like receptor 4 (TLR4) switched to M1 phenotype. The switch can occur by direct activation of M2a MΦs, or indirectly through activation of mesenchymal stem cells. M1 MΦ-derived exosomes activated NFкB to reverse quiescent BCCs to cycling cells. Using an in vivo model of BC dormancy, injected Mi MOs sensitized BCCs to carboplatin and increased host survival. In summary, we have shown how BM stromal MΦs, through exosomes, regulate the behavior of BCCs, by either inducing or reversing dormancy.

## Introduction

Breast cancer (BC) cells (BCCs) may exist in cellular quiescence (dormancy) for decades^1,2^. Disseminated BCCs can enter the bone marrow (BM) long before detection^3,4^. This allows for the establishment of BC dormancy before clinical diagnosis, in addition to transition into cellular quiescence during the clinical course of the disease^5–7^. As compared to micrometastasis in sentinel lymph nodes, BC metastasis to the BM leads to a worse prognosis^8^. BM stromal cells form a critical niche for BCCs to survive. The stromal cells facilitate BCC quiescence, immune escape, changes in cytokine production and gap junctional intercellular communication (GJIC)^9,10^.

Precise targeting of dormant BCCs in BM is a challenge. The quiescent BCCs have stem cell-like properties, and share similarities with endogenous hematopoietic stem cells (HSCs). The anatomical location of the cancer cells with HSCs makes it difficult to target the dormant BCCs without untoward effects on the hematopoietic system^10^. Nonetheless, an understanding of how BM stroma support BCC dormancy is important since the same stromal cells can also cause BC resurgence^11–13^. BM stroma is comprised of several cell types such as macrophages (MΦs), fibroblasts, osteoblasts, mesenchymal stem cells (MSCs), and adipocytes^13,14^.

MΦs are broadly divided into nonactivated, classically activated (M1) and alternatively activated (M2) types^15–17^. M2 MΦs are classified as M2a, M2b, M2c, or M2d and such designation, depends on the mode of activation^16^. M1 MΦs elicit a proinflammatory response and M2 MΦs, immune suppression, wound healing, and angiogenesis^17^. The biological function of a particular MΦ type may be influenced by the surrounding niche, such as MSCs within BM^14,18^. We tested the hypothesis that activation of stromal cells causes one of its component, M2 MΦ, to polarize into the M1 phenotype to reverse dormant BCCs into proliferating cells.

This study activated toll-like receptor 4 (TLR4) on MΦs to study how this influence BC behavior because TLR4 has been linked to cancer recurrence^19–21^. TLR4 is a member of the pattern recognition receptor (PRR) system, which can be stimulated by microbiome-derived ligands such as lipopolysaccharide (LPS). TLR4 can also bind to other pathogen associated molecular pattern and endogenous damage-associated molecular patterns (DAMPs)^22^.

We report on conversion of M2 MΦs into M1 MΦ phenotype by LPS. Such conversion occurred directly on M2 MΦs and indirectly, through MSCs. The M1 MΦs secrete exosomes, which reversed the quiescent phase of BCCs, particularly the cancer stem cell (CSC) phenotype without affecting their “stemness”^10^. In the presence of M1 MΦs, the majority of otherwise chemoresistant CSCs were responsive to carboplatin. Injection of M1 MΦs into immune deficient mice harboring dormant BCCs reversed dormancy resulting in the BCCs becoming sensitive to carboplatin. The mice injected with M1 MΦs showed prolonged survival with no evidence of the dormant BCC. In contrast, mice injected with M2a MΦs survived, but with persistence of the dormant BCCs. The data are discussed in the context of how BCCs may react to PRR stimulation, and the potential risk for long-term BC survivors.

## Materials and methods

### Ethical statement

The use of human blood and BM aspirates was approved by the Institutional Review Board (IRB) of Rutgers, Newark Campus. All subjects ranged were 18–35 years and signed the informed consent forms. The Institutional Animal Care and Use Committee (IACUC) approved the use of mice. The studies presented in this study adhered to the approved protocols.

### Culture of BM stroma/assessment of MΦ component

Stroma was prepared from BM aspirates as described^23^. Total BM aspirate with nucleated cells (10^7^) were added to 25-cm^2^ tissue culture flasks (Falcon 3109) in stromal media. At day 3, the red blood cells and neutrophils were removed by Ficoll–Hypaque density gradient and the mononuclear cells retrieved from each flask were re-added in fresh stromal medium. The flasks were incubated until confluence with a weekly replacement of 50% fresh stromal medium.

Confluent stromal cells were assessed for MΦ phenotype by immunofluorescence using anti-CD206 (M2) and anti-MHC-II (M1). MΦs are trypsin resistant and de-adherence requires treatment with 0.2% collagenase^24^. The recovered MΦs were incubated in Teflon jars for 2 h to allow any degraded membrane protein to be re-expressed. In five analyses with stroma from a different donor we observed 10 ± 1.3% (±SD) CD206+/MHC-II-cells. Indeed, MΦs, which are part of the stromal compartment, are important for functional hematopoiesis^25^.

### Monocyte/MΦ culture

The culture of M1 and M2a MΦs was performed as described using human peripheral blood mononuclear cells (PBMCs) and BM mononuclear (BMNCs)^26–28^. Monocytes were selected by incubating the mononuclear cells with Dynabeads Flow Comp human CD14^+^ kit from Invitrogen (Carlsbad, CA). This resulted in cells that were positive for nonspecific esterase and >99% CD14+ cells by flow cytometry (Fig. S1). The monocytes (2 × 10^6^ cells/mL) were incubated for 48 h in 6-well plates with RPMI 1640 containing 10% FCS and 50 ng/mL of M-CSF^29^. The media were replaced with fresh media containing 10 ng/mL IFNγ for M1 MΦ and 20 ng/mL IL-4 for M2a MΦ. MΦs within BM stroma were ascribed the designation of M2 and were CD206+ and MHCII−.

### Cell synchronization

BCCs were synchronized for 72 h in serum-free media supplemented with 1× insulin–transferrin–selenium. At synchronization, the cells were challenged with exosomes by replacing the media containing 10% FCS.

### Cell health

The assessment of live/dead cells, referred as cell vitality, used two different dyes from Molecular Probes. The live cells can convert a redox dye (resazurin) into a fluorescent end product (C12-resorufin). SYTOX green dye is a permeable cell stain that is used to measure either injured or dead cells. Cells were incubated with 500 nM C12-resazurin and 10 nM SYTOX in 100 µl 1× phosphate-buffered saline (PBS) for 15 min at 37 °C. Cell suspension was diluted with 400 µl 1× PBS. Stained cells were immediately analyzed on FACSCalibur (excited at 488 nm and measured fluorescence emission at 530 and 575 nm). Cell populations were gated into three groups—live, injured, and dead, and then quantified.

### Exosome isolation and characterization

Exosomes were isolated using a multi-step process. Large vesicles were eliminated by sequential centrifugation up to 50,000*g* as described^12^. The remaining particles were pelleted by ultracentrifugation (Sorvall mTx 150, Thermo Scientific, Springfield, NJ) at 100,000*g* for 18 h. Vesicles expressing CD63 were immunoselected using CD63 magnetic bead isolation. The recovered particle size was verified by nanoparticle tracking analysis (NTA) using a NanoSight NS300 instrument (Amesbury, UK) as described^11^. The data were analyzed with the NTA software (NANOSight version 2.3) using dilutions with deionized water. Three videos at a minimum of 200 completed tracks were collected at 30-s time intervals/video per sample.

Exosomes were further distinguished by flow cytometry with magnetic beads coupled to anti-CD9-PE, anti-CD63-FITC and anti-ALIX-APC (BD Biosciences).

### In vivo studies for roles of M1 and M2 MФs

BALB/c nude female mice (6 weeks) were purchased from Taconic Farms (Germantown, NY) and housed in an AAALAC-accredited facility. BC dormancy in BM was established by tail vein injection of Oct4^hi^ MDA-MB-231 or T47D (10^3^ in 0.2 mL 1× PBS), as described^10^. To ensure dormancy mice were injected intraperitoneally with low dose of carboplatin (2 mg/kg) at days 2 and 5, resulting in Oct4^hi^ BCCs in the endosteal regions of femurs. At day 7, the mice were divided into groups of ten, and injected intravenously with (a) 10^6^ CMTMR-labeled M1 MΦ in 0.2 ml 1× PBS; (b) 10^6^ CMTMR-labeled M2a MΦ in 0.2 mL 1× PBS; (c) Control, injected with 0.2 mL 1× PBS. Each experiment was repeated three times. At days 10, 12, and 15, the mice were injected with 5 mg/mL carboplatin. At day 20, the mice were euthanized and the femurs and lungs were harvested. The femurs were washed to remove the cells and the endosteal region scraped and immediately analyzed for fluorescent cells. Femurs were also decalcified, embedded in paraffin and then sectioned at the digital imaging and histology core facility at New Jersey Medical School. Sections from the femurs of mice were subjected to immunohistochemistry for M1 and M2 MФs (see Supplemental method) In separate studies, survival curve was done for 30 days in three experiments, each with groups of 10 mice.

### Anti-miR-222 and -223 transfected MSCs

MSCs were co-transfected with anti-miR-223 and anti-miR-222 or negative control anti-miR using Lipofectamine RNAiMAX reagent (Life Technologies Invitrogen, Carlsbad, CA) as described^11^. The anti-miRs were purchased from Life Technologies—Applied Biosystems (Grand Island, NY). The MSCs were washed and then resuspended in PBS for injection into nude mice.

### In vivo studies for MФ type between tumor-free and tumor-bearing mice

BC dormancy was established in female BALB/c nude mice (6 weeks) old as described, also explained above^10^. Another group was injected with PBS for the purpose of assessing MФ type in femurs. The dormant group was further subdivided into two groups: Group 1 was injected 10^6^ MSCs transfected with anti-miR222/223 and Group 2, with control anti-miR. The anti-miRs can enter the dormant BCCs to reverse dormancy into proliferating BCCs^11^.

### Statistical analyses

The data were analyzed using one-way ANOVA with Bonferroni’s multiple comparison test as the post hoc test or two-way ANOVA with Bonferroni correction. A *p* value ≤ 0.05 was considered to be significant. The significant effects of exosomes and media were deduced by *p* values of <0.001 in two-way ANOVA with Bonferroni post-test that compared cycling progression, G0/G1 vs. S-phase.

## Results

### BM stromal M2 MΦs to M1 type

**Fig. 1:**
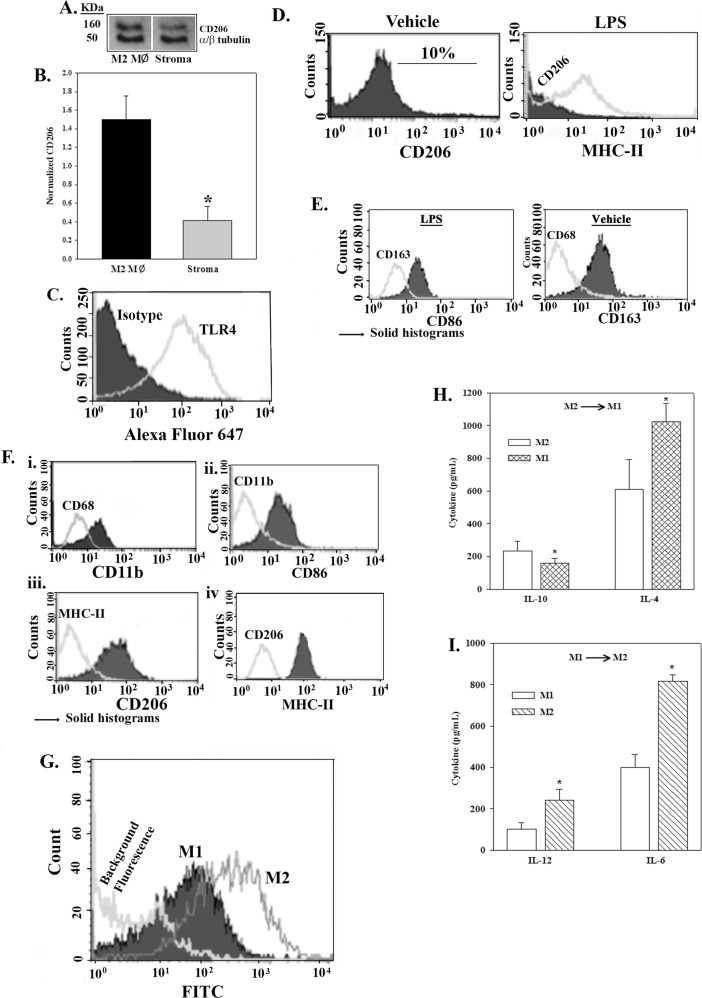
Behavior of MΦs within BM stroma. **a** Representative (*n* = 3) western blot for CD206 and a/b tubulin with whole cell extracts from three different donors of stroma or purified M2a MΦ. **b** The band densities for CD206 were normalized using the bands for a/b tubulin. The results are presented as the mean ± SD, *n* = 3. **c** Representative (*n* = 3) flow cytometry for TLR4 with BM stroma stimulated with 10 ng/mL LPS. **d** Representative (*n* = 3) histogram for CD206 and MHC-II with stromal cells stimulated with vehicle or 10 ng/mL LPS, respectively. The right panel shows the overlay of CD206 and MHC-II. **e** Stromal cells (*n* = 3) were stimulated with 10 ng/mL LPS or vehicle. The cells were removed with 0.2% collagenase followed by a 2-h incubation to allow cell surface proteins to recover. The CD14+ cells were gated by flow cytometry and then analyzed for CD68 and CD163. The histograms are shown as overlays with the solid graphs representing the label on the *x*-axis. **f** PBMC and BMMC-derived M2a and M1 MΦs were studied for CD11b and CD68. (i–ii) The histogram were overlaid and the solid graphs are indicated by the *y*-axis. The M2a MΦs were treated with vehicle (iii) or LPS-stimulated MSCs (iv). After 16 h, the cells were labeled for CD206 and MHC-II and then analyzed by flow cytometry. The histograms for each were overlaid and the markers shown in the solid graphs are placed on the *y*-axis. **g** M1 and M2 MΦ were studied for phagocytic properties by incubating with IgG-FITC coupled to beads. The cells were washed and then analyzed by flow cytometry for FITC. Shown is a representative overlay of three independent experiments. **h** MΦs in cultures with activated MSCs were negatively selected and then studied for IL-10 and IL-4 levels, ±SD (pg/mL), *n* = 4. **I** M1 MΦs from “H” were retained for 24 h to re-repolarize to M2a MΦ and then the media analyzed for IL-12 and IL-6. The results are expressed as the mean levels (pg/mL) ± SD

In order to include all components of BM stroma to understand BC dormancy, nonhematopoietic cells and MΦs, stromal cultures are established with BM aspirates^10,30,31^. Western blots with whole cell extracts identified the marker of M2 MФs, mannose receptor C-type1 (MRC1/CD206), within stroma, similar to extracts from M2a MΦ control (Fig. 1a)^32^. The normalized band densities showed CD206 significantly (*p* < 0.05) reduced in stroma as compared to control M2a MΦs (Fig. 1b), consistent with ~10% M2 MΦs in stromal cultures, the literature and also, labeling of murine femur for CD206 (11 ± 2%, *n* = 5)^13,33^.

Stromal M2 MΦs could be switched to M1 phenotype as indicated by activation of TLR4 with LPS^34,35^ (Fig. 1c). Flow cytometry confirmed 10% CD206+ cells in unstimulated stroma (Fig. 1d, left panel) and 10% M1 MΦs (MHC-II+) with LPS (Fig. 1d). The activated MΦs expressed CD86 and reduced CD163 whereas unstimulated/vehicle MΦs showed the reverse (Fig. 1e). Since CD163 is expressed on endogenous BM MΦ, our model recapitulated occurrences in BM^36^. The switch of MФs to M1 type was confirmed M1 by cytokine production using purified MΦs (Fig. 1h, i). Together the findings showed conversion of stromal M2 MΦs into M1 type.

### Indirect polarization of M2a MΦ to M1 type via MSCs

Since direct activation of TLR4 on M2 MΦs switched to M1 type (Fig. 1a–e), we next asked if this can occur indirectly by MSCs^11,37^. M2a and M1 MΦs from PBMCs or BMNCs of healthy donors expressed CD11b and CD86, respectively (Fig. 1f, i–ii). LPS- (stimulated) or vehicle- (unstimulated) MSCs, added to M2a MΦs for 16 h, indicated no change in MΦ phenotype by unstimulated MSCs (CD206+) whereas stimulated MSCs switched the MΦs to M1 phenotype (MHC-II+) (Fig. 1f, iii–iv). The MSCs did not affect the MΦ function, based on phagocytosis of fluorescence latex particles (Fig. 1g). The switch to M1 MΦs were validated by higher IL-4 level as compared to M2a MΦs (Fig. 1h). In summary, this section showed indirect polarization of M1 and M2a MΦs by MSCs. The mechanisms by which MSCs control MΦ types is included in a separate publication.

### Cytokine production by M1 and M2 MΦs

In order to validate the flow cytometry analyses in M2–M1 MΦ conversion, we performed ELISA for associated cytokines^38^. M2 to M1 MΦ conversion caused a significant (*p* < 0.05) decrease of IL-10 whereas M1 to M2 MΦ increased IL-6 and IL-12 (Fig. 1h, i). The results indicated functional plasticity by M1 and M2 MΦs, consistent with the literature^38^.

### Discerning MФ type in tumor and nontumor bearing femurs

**Fig. 2:**
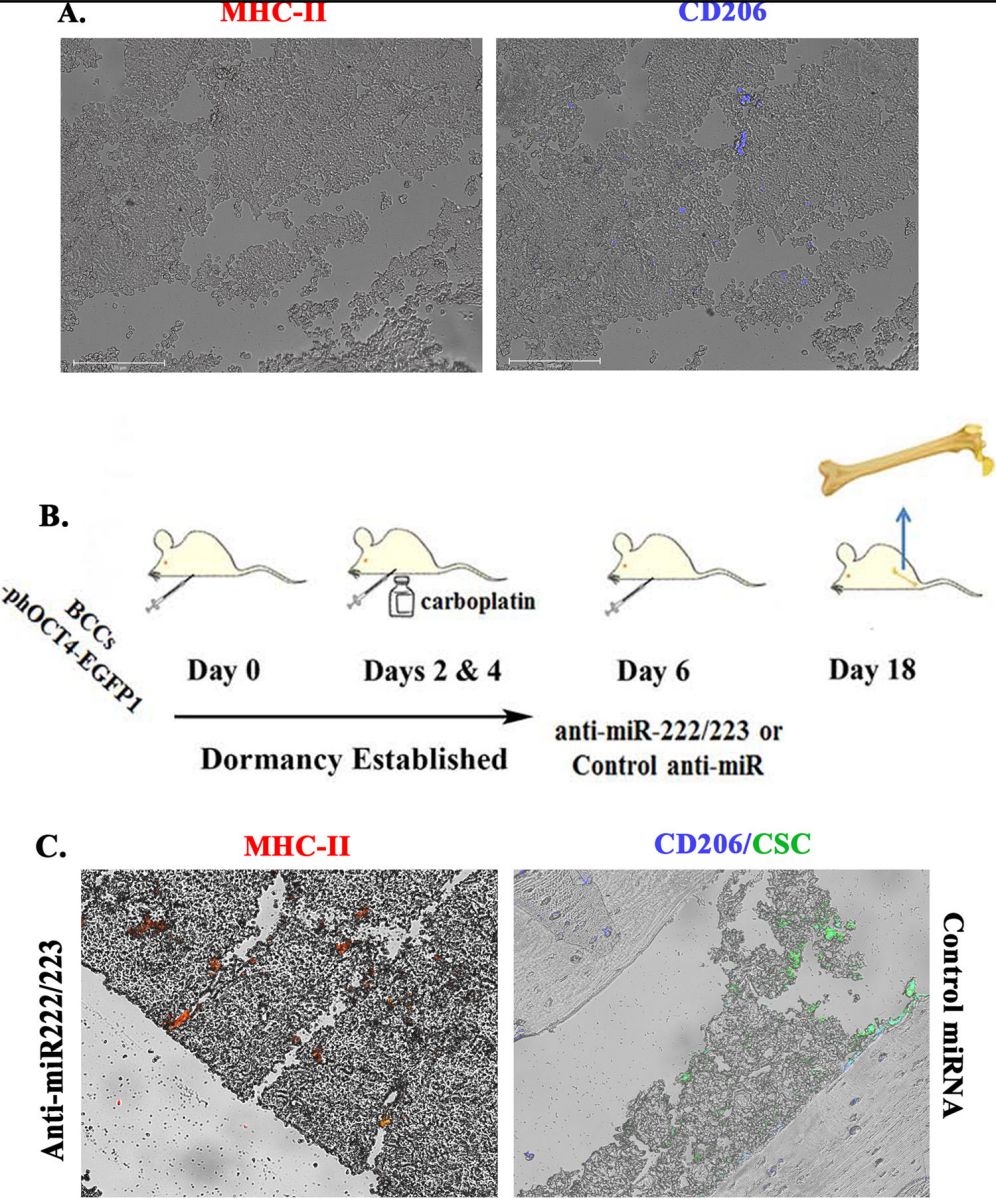
MФ type in BALB/c femurs and with dormant and reverse dormant BCCs. **a** Femurs were subjected to immunohistochemistry for MHC-II using indirect labeling with secondary Texas Red IgG (left panel) and for CD206 with Alexa Fluor conjugated secondary IgG (right panel). **b** Dormant BCCs were established in female BALB/c. Dormancy was assured by injected the mice with 5 mg/kg of carboplatin at Days 2 and 4. At Day 6, the mice were injected with MSCs transfected with anti-miR222/223 previously shown to reverse dormancy. At day 18, the mice were euthanized and the femurs harvested. **c** Sections from the femurs in “B” were analyzed for MHC-II and CD206. The green fluorescence cells in the right panel represents CSCs. The images represents five independent sections, each from a different mouse

This set of studies validated the predominance of M2 MФs in stromal cultures (Fig. 1). We first analyzed the femurs of nude female BALB/c mice by immunohistochemistry for M1 MФs (MHC-II) and M2 MФs (CD206). Since the nude mice are deficient in T-cells and the mice were not activated immunologically, the MHC-II could be a surrogate of M1 MФ. We did not detect MHC-II but CD206 was detected, verifying M2 MФs in mice femur (Fig. 2a).

Next, we studied the BALB/c mice femurs for MФ type using models with dormant BCCs and reverse dormancy when the mice was treated with anti-miR222/223^11^ (Fig. 2b). M1 MФs were detected during reverse dormancy (Fig. 2c, left panel) whereas those with dormant BCCs (GFP+) showed cells positive for CD206 (blue) (Fig. 2c. right panel). In summary, M2 MФs were present in nude BALB/c femurs with dormant BCCs and M1 type during reverse dormancy. We estimated about 5–10% M2 MФs in the femurs, close to the endosteal region.

### Relative efficiencies in GJIC by M1 or M2 MΦs with CSCs

**Fig. 3:**
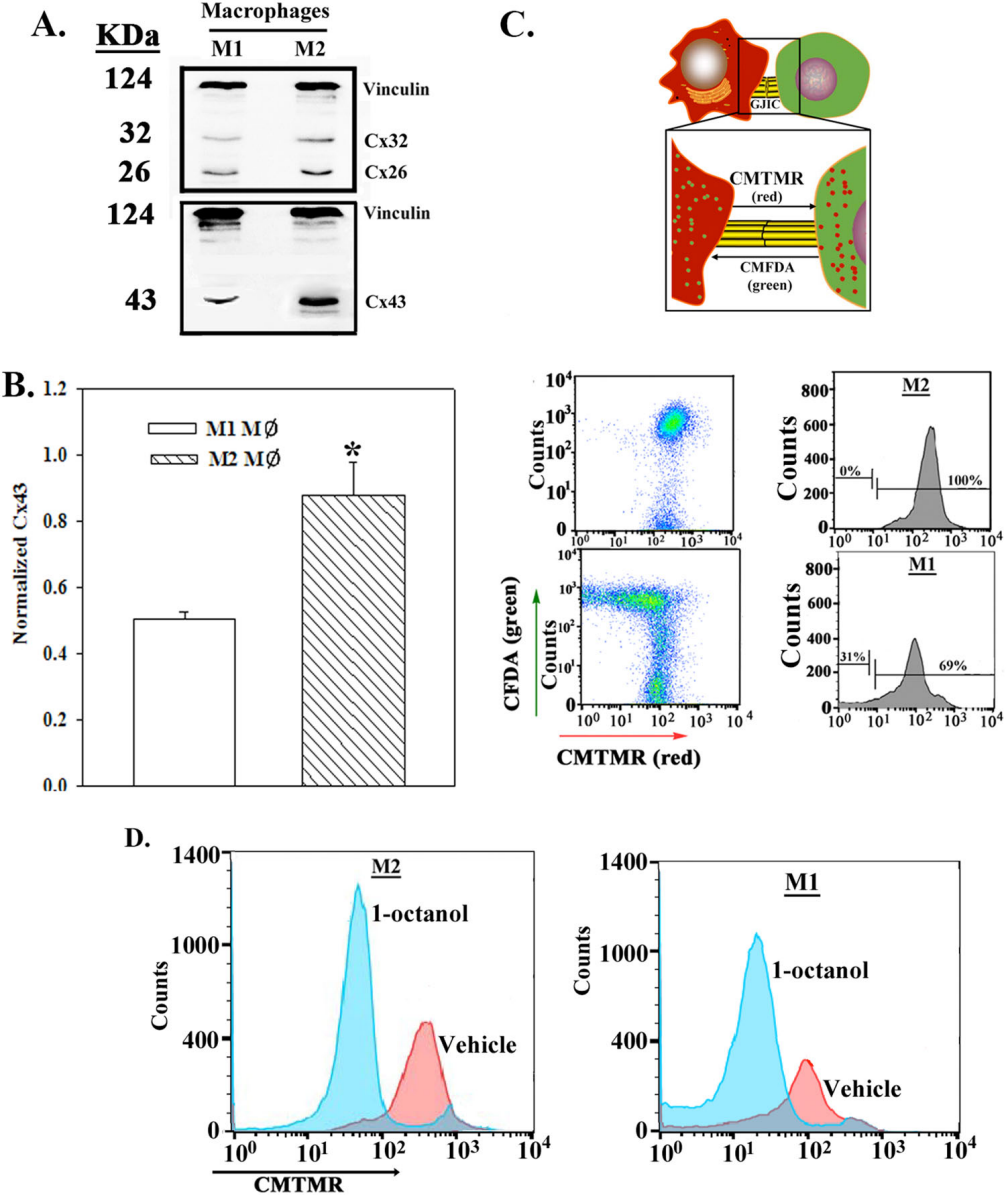
GJIC between CSCs and M1/M2a MΦs. **a** Western blot for Cx26 and Cx32 (top panel) and Cx43 (bottom panel) with extracts from BMNC-derived MΦs. **b** The densities for the top bands (total Cx43) were normalized with bands for Vinculin and then presented as the mean ± SD, *n* = 3. *p < 0.05 vs. M1 MΦ. **c** Top panels show the experimental design in which the CSCs were green due to Oct4a-GFP. The CSCs were labeled with CFDA (green) and the MΦs with CMTMR (red). The M2 MΦs (middle panels) or M1 MΦs (third panels) were co-cultured at 1:1 ratio with 10^6^ CSCs from MDA-MB-231 or T47D in 6-well plates. After 24 h, the CSCs were selected by trypsin digestion and then examined for CMTMR transfer by flow cytometry. Representative histograms and scatter plots are shown for MDA-MB-231. **d** The studies in “C” were repeated in the presence of 300 µM 1-octanol or vehicle

We reported on sustained BC dormancy by GJIC between BM stroma and CSCs^10^. In order to determine if M2 MΦs can contribute to GJIC, we negatively select M1 and M2 MΦs within BM stroma and then analyzed the extracts for Cx26, Cx32, and Cx43 by western blots. Band densities for Cx32 and Cx43 were significantly (*p* < 0.05) higher for M2 MΦs and similar for Cx26 (Fig. 3a, b).

The function of Cxs were studied in a two-way dye transfer assay between MΦs and BCC subsets in the presence or absence of a pharmacological agent (1-octanol) (Fig. 3c, top panel). The CSCs were green due to pOct4a-GFP and were labeled with CFDA (green). MΦs were labeled with CMTMR (red). Flow cytometry for CMTMR transfer in the GFP cells (CSCs) indicated transfer from M2 MΦ to CSCs, which was significantly reduced with M1 MΦs (Fig. 3c). The dye transfer was specific based on a blunting effect by 1-Octanol, which retained cell viability (Fig. 3d and S2).

### Distinct effects by M1 and M2a MΦ on BCC cycling

**Fig. 4:**
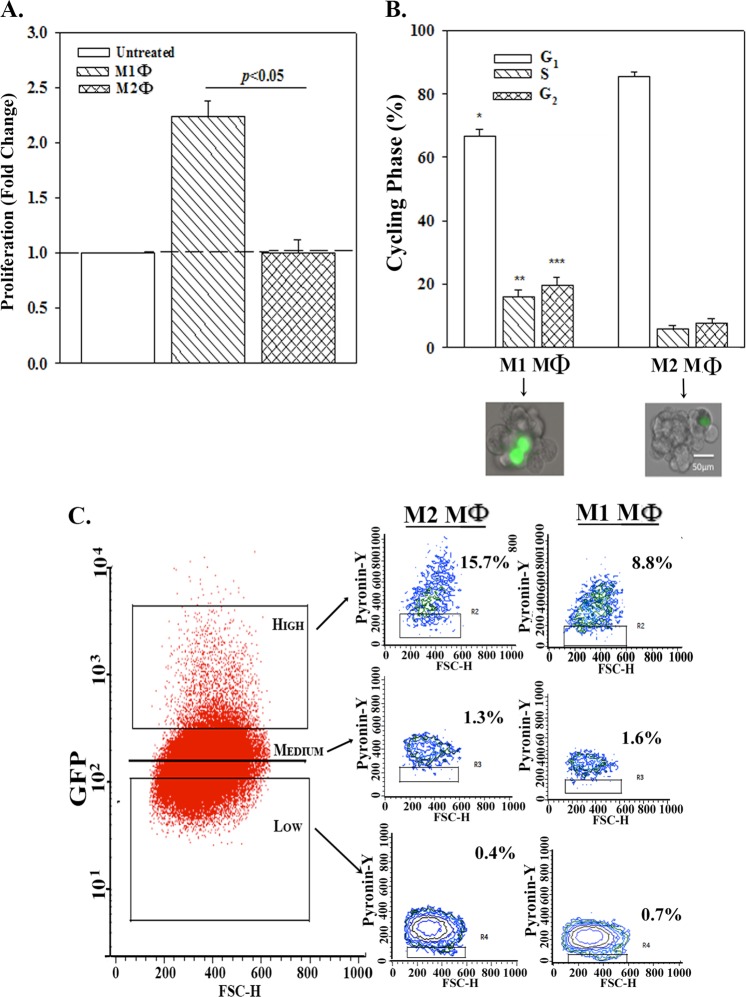
Increased proliferation and cell cycling of BCCs by M1 MΦ. **a** BCCs were co-cultured with M1 or M2 MΦs at 1:1 ratio or alone at 10^6^ cells each in T75 tissue culture flasks. After 72 h, cell proliferation was assessed by Cyquant Assay. The data with BCCs alone are normalized to 1 and the experimental expressed as fold change (±SD, *n* = 4). **b** The studies in “A” were repeated with BCCs stably transfected with pOct4a-GFP. After 72 h, the BCCs were analyzed for cell cycle by PI labeling. The data for cells in G1, S, and G2 phases are presented as the mean ± SD, *n* = 4. Tumorspheres from the pOct4a-GFP BCCs in cultures with MΦs were cultured to form spheres by limiting dilution. Images at 200× magnification with the Evos fl imager are shown below. **c** The studies in “B” were repeated except the cycling analyses were done with 7-AAD and Pyronin Y labeling. Left panels show the gating scheme for the analyses based on relative GFP intensities. The cells with low-DNA content were further analyzed for low RNA to represent cells in G0 phase. *p < 0.05 vs. cultures with M2a MΦ in G1 phase. **p < 0.05 vs. cultures with M2a MΦ in S phase. ***p < 0.05 vs. cultures with M2a MΦ in G2 phase

Since M1 MΦs showed less efficiency in GJIC with CSCs, we asked if M1 MΦs can reverse CSC quiescence. Equal numbers of unsorted BCCs and M1 or M2a MΦs (from BMNCs), co-cultured for 72 h, showed significantly (*p* < 0.05) increased BCC proliferation as compared to M2a MΦs (Fig. 3a). Gating of the CSC subset (bright GFP)^10^, we noted a significant (*p* < 0.05) decrease in G1 phase and increase in S/G2 phases by M1 MΦs (Fig. 4b and Fig. S3). The effects were specific for MΦs since the major stromal cells (fibroblasts) did not affect the cycling of BCCs (Fig. S4).

Limiting dilution of tumorspheres from CSCs (Oct4 high) in 72-h co-culture with M1 or M2 MΦs indicated no effect on self-renewal after fiver serial passages. Representative spheres are shown from passage 5 (Fig. 4b, lower panels).

### Effects of M1 and M2 MΦs on the cycling of BCC subsets

The effect of MΦs on cell cycle of distinct BCCs subsets was assessed with Oct4A-GFP transfectants^10^ (Fig. 4c, left panel). The low-DNA/non-cycling cells (7-AAD) were analyzed for low RNA (Pyronin Y) to discern G_0_ and G_1_ phases. The CSCs (GFP^hi^) were mostly in G_0_ phase with M2a MΦs (Fig. 4c, middle and top panel, 15.7%). M1 MΦs significantly (*p* < 0.05) reduced the percentage of G_0_ cells (Fig. 4c, right and top panel, 8.8%). The other subsets were not affected regardless by the MΦ type (Fig. 4c, lower two panels). In summary, the results indicated that the CSCs were mostly affected by M1 and M2 MΦs with respect to their cycling phase.

### Effects of MΦ-derived exosomes on the cycling phase of BCCs

**Fig. 5:**
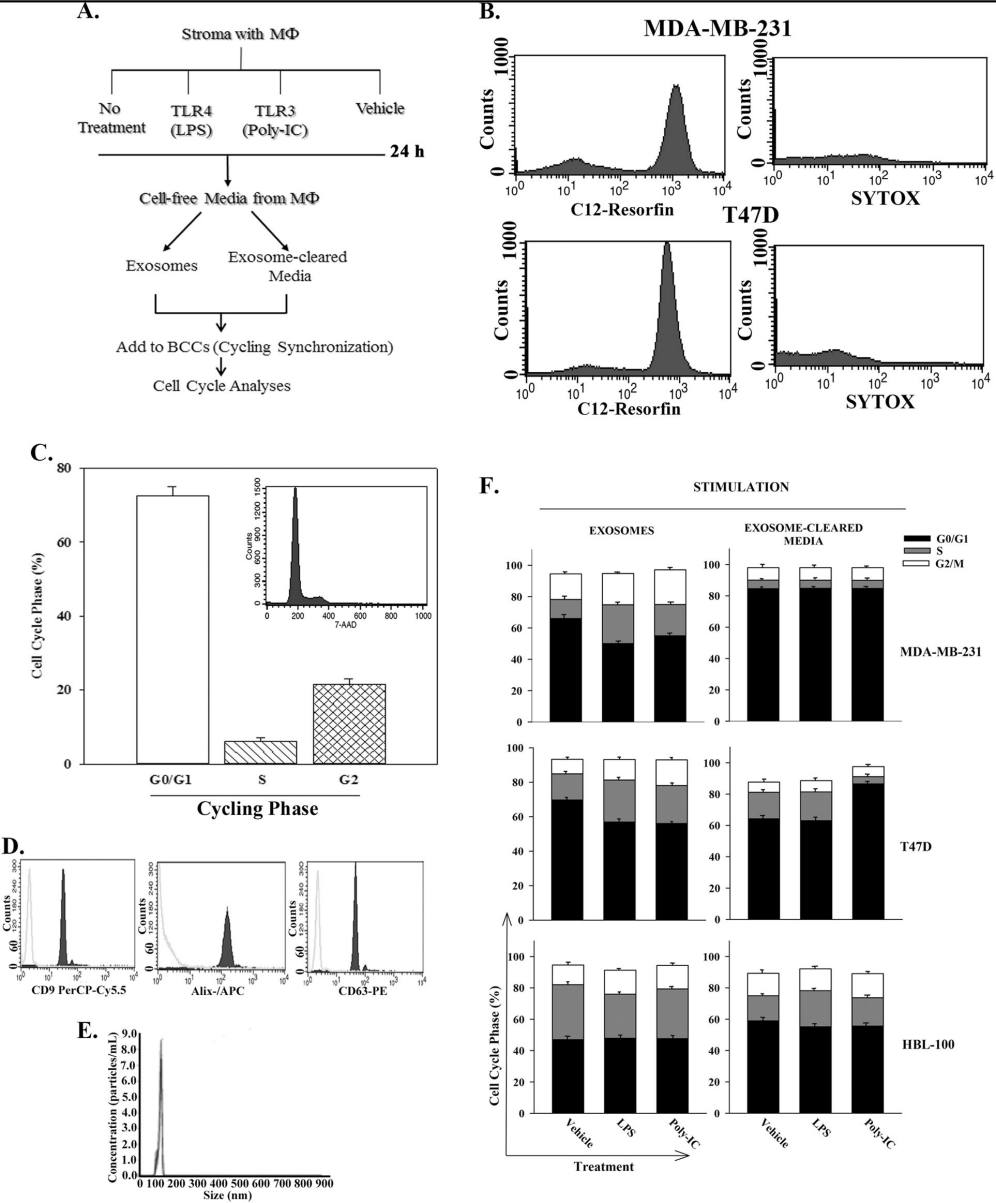
Exosomes from MΦs in the behavior of quiescent BCCs. **a** The diagram shows the overall experimental design used for the data in this figure. **b** The serum-starved/synchronized BCCs were assessed for cell health as described in the Materials and Method section. **c** Cell cycle analyses were performed with the serum-starved BCCs. Inset shows a representative histogram of results of MDA-MB-231 with PI labeling. The results are shown for percent cells in different cycling phase, mean ± SD, *n* = 6. **d** A representative histogram of flow cytometry for tetraspanin proteins for the isolated exosomes. **e** A representative graph of the NTA is shown for the isolated exosomes. **f** Stacked bar graphs of cell cycle analyses by PI labeling of serum-starved BCCs treated for 24 h with MΦs-derived exosomes. The MΦs were isolated from stroma and then treated with vehicle, 10 ng/mL LPS and 10 ng/mL poly-IC as outlined in “A”. The results are mean% cells in cycling phase, ±SD, *n* = 7

Figure 5a outlined the approach that was used to analyze the role of MΦ secretome on BCC cycling phase. Stroma was activated with 10 ng/mL LPS (TLR4), 10 ng/mL Poly-IC (TLR3) or vehicle, and after 24 h the MΦs were negatively selected by trypsin. The stimulated MΦs expressed MHC-II (M1) and unstimulated, MHC-II-/CD206+ (M2). The purified MΦs were re-incubated in exosome-free media. After 24 h, the media were collected and then fractionated into exosome and exosome-cleared media. Exosomes (4 × 10^7^, per dose–response, Fig. S5) and the cleared media were added to 10^6^ serum-starved BCCs, which were mostly in G_0_/G_1_ phase and good health (Fig. 5b, c). The exosomes were <120 nm and expressed tetraspanin proteins (Fig. 5d, e).

The cycling phase of nontumorigenic HBL-100 cells were not influenced by the exosomes or cleared media (Fig. 5f). However, exosomes from activated MФs (M1) reduced the cycling quiescence of BCCs (Fig. 5f, left panels). This effect was specific to exosomes since 10% FCS showed no change (Fig. 5f, right panels). The results indicated that exosomes from activated MΦ excited BCCs from cycling quiescence.

### MΦ-derived exosomes in BCC migration

**Fig. 6:**
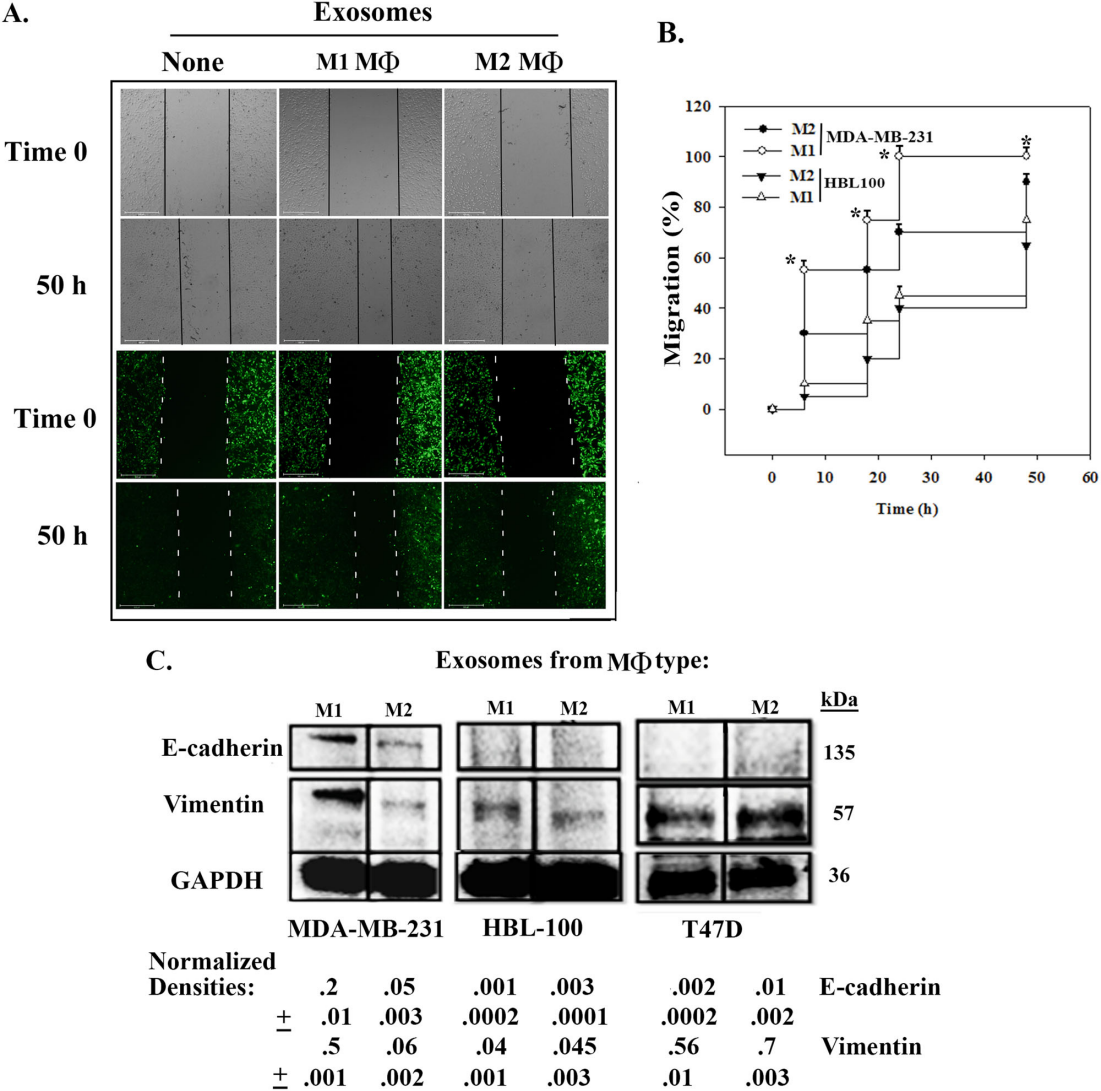
Migration of CSCs exposed to MΦ-derived exosomes and EMT/MET proteins in the treated CSCs. **a** CSCs from MDA-MB-231 were incubated with exosomes from M1 or M2 MΦs. Control cultures did not contain exosome. Images are shown for Time 0 (time of scratch) and for 50 h later. Top panels show the visible images and the bottom, GFP cells representing CSCs. **b** The % migration from of the scratch assay performed as for “A” with CSCs from MDA-MB-231. Parallel analyses were performed with the nontumorigenic HBL100. The data from both cell lines are plotted with each point representing the mean ± SD of four independent experiments. **c** Whole cell extracts from BCCs (MDA-MB-231 and T47D) and the nontumorigenic cell, HBL-100, were analyzed by western blots for E-cadherin and vimentin. The bands were normalized with GAPDH and shown below, mean ± SD, *n* = 3. * *p* < 0.05 vs. M2 exosomes from MDA-MB-231

We determined if M1-mediated cycling transition of CSCs correlates with migration and epithelial/mesenchymal transition (EMT)/MET. Migration using scratch assay with CSCs, mostly in G_0_/G_1_ cycling phase^10^ and, treated with 2 × 10^7^ exosomes from MФs (M2 or M1) or without exosomes. A representative image for MDA-MB-231 is shown for time 0 up to 50 h (Fig. 6a). The plot (mean ± SD, *n* = 4) of the scratch assay indicated a significant (*p* < 0.05) increase in migration with exosomes from M1 as compared to M2 (Fig. 6b).

Western blots for vimentin and E-cadherin were increased in MDA-MB-231 exposed to exosomes from M1 and M2 MΦs. This suggested mixed behavior of BCCs with respect to EMT and MET (Fig. 6c). There was no marked change for T47D and HBL-100 cells.

### Exosomes from M1 MΦs enhanced BCC cycling via NFкB

**Fig. 7:**
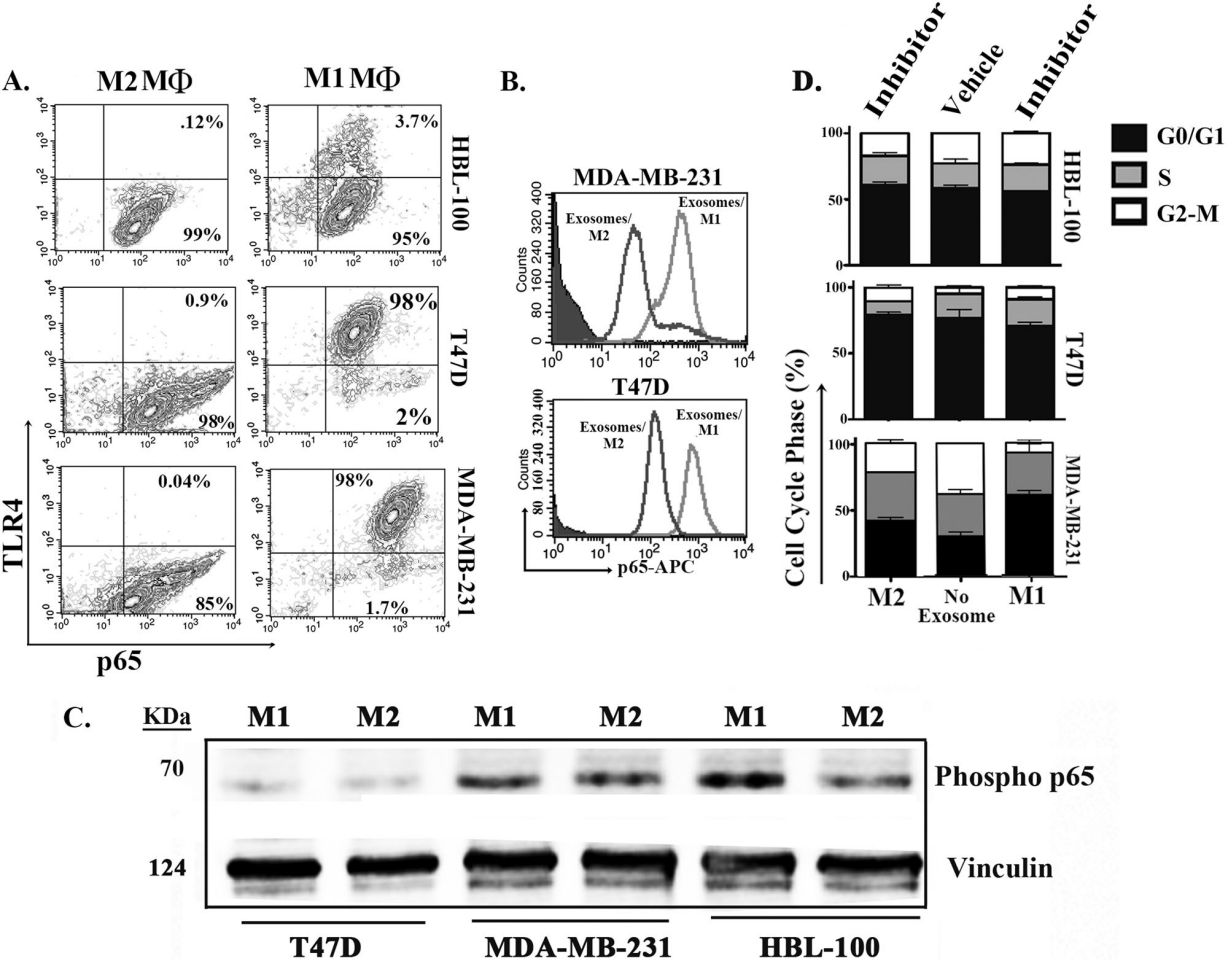
TLR4 and p65 in serum-starved BCCs **a** BCCs (MDA-MB-231 and T47D) and the nontumorigenic cell line, HBL-100, were synchronized as shown in Fig. 5a. The cells were treated with exosomes from stroma-derived M1 or M2 MΦ. After 24 h, the cells were analyzed by flow cytometry for p65 and TLR4. Representative scatter plots are shown for three independent experiments. **b** The data from “A” are presented as the mean fluorescence intensities for p65. **c** The cell extracts from “A” were analyzed for phospho-p65 by western blots and normalized for vinculin. **d** Synchronized BCCs and the non-tumorigenic HBL-100 cells were treated with 20 μM of PDTC for 18 h. After this, the cells were exposed to exosomes collected from stroma-derived MΦs (M1 or M2), or no exosome. After 24 h, the cells were analyzed for cell cycle by PI labeling. The stacked graphs were established for three independent experiments

Active NFκB can regulate cell proliferation, survival, and tumor progression^39–41^. We asked if M1-derived exosomes can induce BCC cycling via active NFκB. Functional studies were needed because phosphorylated (phospho) p65 in the cytosol may not be active^42^. Serum-starved BCCs and nontumorigenic HBL-100 cells were treated with exosomes from M1 or M2 MΦs. Flow cytometry showed increase of p65 in all cell lines but only M1 MΦ-derived exosomes induced TLR4 on the BCCs (~98% vs. <4% on HBL-100) (Fig. 7a), suggesting that increased TLR4 was endogenously induced and not transferred by exosomes. The data shown as average mean fluorescence intensity (MFI) verified p65 increase in cells treated with M1-derived exosomes (Fig. 7b). To further verify NFκB activation, we analyzed the cell extracts for phospho-p65 and showed increases in band intensities when the BCCs were treated with M1-derived exosomes (Fig. 7c).

The role of p65 in cell cycle behavior of BCCs was studied by pretreating the synchronized cells with 20 µM of NFκB inhibitor (pyrrolidine dithiocarbamate, PDTC) for 24 h^43^. Exosomes from M1 or M2 MΦs were added and 24 h later, the cells were analyzed for cycling phase by PI labeling. PDTC only blunted cycling of M1-derived exosomes for MDA-MB-231 (Fig. 7d), suggesting a different mechanism for low-invasive/metastatic T47D.

### MΦ-derived exosomes on carboplatin sensitivity

**Fig. 8:**
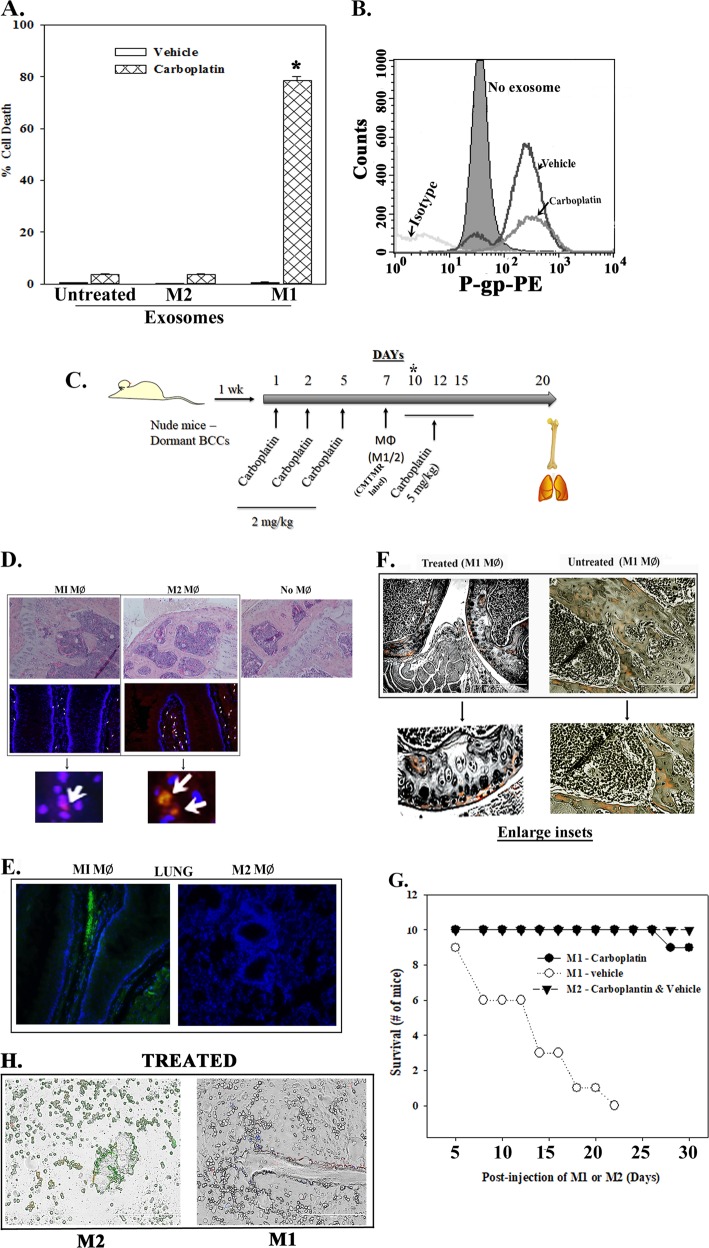
Influence of M1 and M2 MΦs on the sensitivity of dormant BCCs to carboplatin. **a** CSCs were cultured with exosomes from stroma-derived M1 or M2 MΦs or without exosomes. After 24 h, the cells were treated with 50 µg/mL carboplatin or vehicle for 72 h and then studied for cell health. The % cell death is presented as the mean ± SD, *n* = 4. *p < 0.01 vs. carboplatin-treated cells with M2 exosomes or untreated. **b** The CSCs treated with exosomes from “A” were studied for P-gp by flow cytometry. **c** A diagram outlining the method used in the in vivo studies with nude female BALB/c mice. The asterisk represents the time when three mice were euthanized to determine if the injected CMTMR-labeled MΦs entered the femurs. **d** Hematoxylin-stained femurs of mice with dormant CSCs, injected with M1 or M2 MΦs, or no MΦ (top panels). The middle panels shows cellular regions labeled with DAPI (blue) and red fluorescent MΦs. The lower panels show a large inset of what the arrows represent in the middle panels. **e** The lungs of mice were examined at day 10 for CSCs (GFP+). Shown are sections labeled with DAPI (blue) and the GFP cells shown in mice injected with M1 MΦs. **f** Sections of decalcified femurs of mice injected with M1 MΦs with or without carboplatin treatment. Shown are only red regions for the treated femurs indicating labeled MΦs. The untreated femurs have yellow (MΦs and CSCs), red (MΦs), and green (CSCs) sections. Below are enlarged insets of the sections. **g** A survival curve for mice (10/group) injected with M1 or M2 MΦs followed by treatment with caroboplatin or vehicle. Since the survival of treated mice injected with M2 MΦs were similar to untreated mice as well as mice without MΦs but given carboplatin, the values were plotted together. **h** Scrapings of the endosteal regions of mice femurs for GFP cells (CSCs) from those injected with M2 (left) and M1 (right) MΦs

Since M1 MФs can transition quiescent BCCs to cycling cells, we asked if the secreted exosomes can sensitize BCCs to carboplatin. This drug allows us to treat all BCCs, irrespective of the hormone status. CSCs, which are in G_0_/G_1_ phase, were incubated with 4 × 10^7^ exosomes from M2a or M1 MΦ, followed by treatment with 50 μg/mL of carboplatin. After 72 h, flow cytometry for the CSC health indicated significant (~80%, *p* < 0.01) cell death by M1 MФs (Fig. 8a). We asked if the resistant ~20% CSCs (Fig. 8a) could be explained by P-gp, which is the protein for the multidrug resistance (MDR1)/ATP binding cassette^10^. Flow cytometry indicated similar MFIs for P-gp in CSCs, exposed to M1 exosomes followed by treatment with vehicle and carboplatin (Fig. 8b). These findings suggested that P-gp expression was dependent on the exosomes and not carboplatin.

### Chemosensitivity of M1 MΦs

Dormant BCCs are carboplatin resistant^10^. Since M1 MΦs caused quiescent BCCs to cycle, we asked if M1 MΦs can be combined with carboplatin to treat dormant BCCs. Using the approach in Fig. 8c, we established dormancy in nude BALB/c and then injected CMTMR (red)-labeled M1 and M2 MΦs^10^. Dormancy was ensured with low carboplatin dose up to day 5. At day 7, the MΦs were injected and 3 days later, mice were treated with higher doses of carboplatin.

Prior to carboplatin treatment, 3 days MΦs injection, we observed no structural change in decalcified hematoxylin-stained femurs (Fig. 8d, top). We also observed migration of CMTMR-labeled MΦ to the femur (red) within the cellular regions (DAPI, blue) (Fig. 8d, row 2—enlarged sections below).

Prior to carboplatin treatment, we observed CSCs (green) in the cellular region of lungs (DAPI, blue) in mice treated with M1 MΦs but undetectable CSCs with M2 injection (Fig. 8e). The latter observation indicated that CSCs within the lungs could not be explained by trapped cells.

At 20 days after the last treatment, we examined scraped sections of femurs with M1 MΦs, treated or untreated with carboplatin. Only red fluorescent cells were noted with carboplatin, indicating elimination of CSCs with Oct4A-GFP (Fig. 8f, left). In contrast, untreated mice showed yellow regions, indicating GFP (injected CSCs) and red CMTMR (MΦs) (Fig. 8f, right). These results indicated that the M1 MΦs enhanced the response of CSCs to carboplatin.

Survival studies (*n* = 10/group) for mice injected with M1 MΦs without carboplatin succumbed by day 22 (Fig. 8g, open circle). The mice with M1 MΦs or M2 MΦs treated with carboplatin survived up to 30 days (Fig. 8g, solid circle and triangle). M2 MΦs maintained the CSCs in dormancy, hence the enhanced survival. Scraped sections of the endosteal region showed GFP(+) cells (injected CSCs) as compared to undetectable CSCs in mice with M1 MΦs and carboplatin (Fig. 8h). In summary, M1 MΦs caused the dormant CSCs to become sensitive to carboplatin whereas M2 MΦs retained dormancy.

## Discussion

This report supports insights on how components of BM stroma can support the clinical and experimental evidence on disseminated BCCs adapting dormancy^44^. BM stroma facilitates dormancy partly through GJIC, miRNA, exosomes, and cytokine regulation^12,45^. This study demonstrates sustained dormancy by M2 MΦs, which could be reversed by M1 MΦs.

Unactivated BM stroma contains M2 MΦs as demonstrated in stromal cultures and, mouse femurs (Figs. 1 and 2). Since inflammation can cause hematopoietic suppression, it is expected that stroma will contain anti-inflammatory M2 MΦs^13^. Our model recapitulated occurrences within BM as CD163+ MΦs are detected in the stromal cultures (Fig. 1)^36^. Similar to GJIC between CSCs and BM stromal cells, we also showed a similar results for M2 MΦs^10,12^ (Fig. 3). GJIC was significantly reduced when M2 MΦs were switched to M1 (Fig. 3). M1 exosomes enhanced CSC migration and also affected the MET/EMT program (Fig. 6). The latter findings are in line with the literature that reported on a dynamic switch between EMT and MET during migration^46^. It is possible that M1 MΦs might revert to M2 and this could result in exosomes from both MΦ types. Regardless, we deduced a complex effect of MΦ-derived cytokines on quiescent BCCs. These findings are consistent with other studies showing a role for M1 and M2 MΦs in the behavior of low-invasive MCF7 cells^47^. The data also supported an indirect role for MSCs in determining the fate of MΦs (Fig. 1f–i).

M1-mediated enhancement of CSC cycling was due to exosomes since the vesicle-free media did not show the same effect (Figs. 4 and 5). Going forward, roles for cytokines need to be examined since there is a potential for technical errors such as degradation of labile cytokines. Also, if there are cytokine receptors on the exosomes, this might absorb the soluble cytokines. A timeline study will account for cytokines with different peak times for rapid autocrine binding to the cognate receptors.

Our findings on M1-derived exosomes to partly explain reversed dormancy are interesting. Although not tested, we speculate that exosomes with different molecular contents from unstimulated MSCs would be important to maintain GJIC between MΦs and stroma. In unpublished studies we have found that CSCs release exosomes that enter stromal cells. The stroma responded by secreting cytokines to support GJIC with the CSCs, hence a method of sustained dormancy. In other unpublished findings, we have observed dedifferentiation of BCCs into cells with stem cell phenotype following exposure of exosomes from BM microenvironmental cells. Thus, the latter findings link exosomes with GJIC since the CSCs show higher efficiency of forming GJIC with other cells in the BM microenvironment^10^. The information discussed in this paragraph are in preparation of manuscripts for publication.

NFкB can be activated by several stimuli and has ubiquitous functions such as regulating cytokine expression. Increased p65 in quiescent BCCs treated with M1 MΦ-derived exosomes was noted for the more aggressive MDA-MB-231 cells. Such treatment was important for the M1 MΦ-mediated cycling of the quiescent cells (Fig. 7). It will be of interest to study other triple negative BCCs to determine if the observed finding is relevant for this BCC subtype. Interestingly, TLR4 was expressed on BCCs after exposure to M1 MΦ-derived exosomes. This finding was of interested to begin other research to understand how dormancy BC might resurge into metastatic disease. As an example, TLR4 can be activated by the release of ligands when the microbiome ligands are released into the periphery. Additionally, TLR4 can be active by DAMPS. These ligands may reverse the dormant phase of BCCs perhaps partly via NFкB activation^48^.

In vivo, M1 MΦs sensitized CSCs to carboplatin (Fig. 8a). The expression of P-gp in BCCs were similar for vehicle and those that survived carboplatin (Fig. 8b). Thus, resistance cannot be explained by P-gp. The mice injected with M1 MΦs and carboplatin showed prolonged survival. We assume that the in vitro studies were inadequate—perhaps requiring additional exosomes from freshly prepared M1 MΦ, or requiring longer treatment. Alternatively, the in vivo outcome suggested that the M1 MΦs might need interaction with other BM microenvironmental cells. Our unpublished data, literature and limited information on MSCs support a MSC-MΦ axis to influence BCC behavior in BM (Fig. 1)^49^. In ongoing research studies, we noted distinct secretomes (soluble and vesicles) by activated and inactivated MSCs. We are currently identifying the candidate secretome(s) involved in determining how the BM microenvironment sustain a particular MΦ type.

It is interest that M1 MΦ-derived exosomes induced TLR4 in BCCs (Fig. 7a). The TLR4 was not exogenously derived from the exosomes because such expression was not noted for the nontumorigenic cells (Fig. 7a). The model incorporating TLR4 and TLR3 was intended to gain insights on BC resurge. TLR4, which is a PRR, can also interact with DAMPs. Thus the incorporation of PRRs in the study is highly significant since these receptors can be activated by the microbiome secretome, which has been linked to BC^50,51^.

Upon synchronization, BCCs showed similar effects on cycling quiescence as the studies conducted with CSCs in the presence of MΦs. This suggested that MΦs may affect any BCC within an environment that promotes survival and reduced cycling. Thus, it is likely that dormant BCCs might be heterogeneous with respect to relative maturity. These findings have added to the general information on BM stromal support of BC survival. The data in this report have provided information that would lead to omics studies for the purpose of understanding how dormancy and cancer recurrence occur. We are in the process of comparing the exosomes from M1 and M2 MΦs. These analyses are insufficient since the preliminary evidence suggest that proteins within the exosomes might also be responsible (Unpublished). The contents of the exosomes involved in these studies are included in an independent paper, in preparation for publication.

We have determined the MΦs in healthy and tumor-bearing mice using a model of dormancy and reverse dormancy^11^ (Fig. 2). M2 and not M1 MФs were detected in the unactivated mice. The M2 MФs continued to be present during dormancy, supporting the findings in this study in which M2 MФs are shown to sustain dormancy. Reversed dormancy, which used cell-delivered miR-222/-223, resulted in a switch in MФs to M1 type. Together, this study showed M2 MФs involvement in dormancy and during recurrence, the MΦ type is switched to M1 type. An unanswered question in this manuscript is the contents of the exosomes that cause the M2 MФs to switch to M1 type to reverse dormancy. We have performed next generation sequencing of the exosomes as well as single-cell sequencing to shown dedifferentiation of BCCs.

## Supplementary information

Supplemental methods and figures
