# Supplementary material for: Exosomes from differentially activated macrophages influence dormancy or resurgence of breast cancer cells within bone marrow stroma
Source: Cell Death Dis. 2019 Jan 25;10(2):59. doi: 10.1038/s41419-019-1304-z (PMC6347644; doi:10.1038/s41419-019-1304-z)
Supplement: Supplementary file 1 — Supplemental methods and figures [file 41419_2019_1304_MOESM1_ESM.docx]

**SUPPLEMENTAL INFORMATION**

**Title:** Exosomes from differentially activated macrophages influence dormancy or resurgence of breast cancer cells within bone marrow stroma.

**Authors:** Nykia D. Walker ^1,2^, Michael Elias ^1^, Khadidiatou Guiro ^1^, Ranvir Bhatia ^1^, Steven J. Greco ^1^, Margarette Bryan ^1^, Marina Gergues ^1,2^, Oleta A. Sandiford ^1,2^, Nicholas M. Ponzio ^3^, Samuel J. Leibovich ^4^, Pranela Rameshwar ^1,2^

^1^Rutgers New Jersey Medical School (NJMS), Newark, NJ, USA. ^2^Rutgers Graduate School of Biomedical Sciences, Newark, NJ, USA. ^3^ NJMS, Dept of Pathology and Laboratory Medicine. and ^4^ NJMS, Department of Cell Biology and Molecular Medicine

**Materials and Methods**

**Reagents**

Tissue culture media, α-MEM, DMEM with high glucose, RPMI 1640, Harris Hematoxylin and Insulin-Transferrin-Selenium were purchased from Thermo Fisher Scientific (Waltham, MA). 1 octanol, Ficoll-Hypaque, bovine serum albumin (BSA), collagenase and lipopolysaccharide (LPS) were purchased from Sigma Aldrich (St. Louis, MO). LPS stock at 1 mg/mL of serum-free DMEM was stored in aliquots at -20°C and then used at a final concentration of 10 ng/mL. This concentration was based on dose-response and time course studies. Total Exosome Isolation Reagent, Exosome-Human CD63 Isolation/Detection Reagent, puromycin and geneticin G418 were purchased from Invitrogen (Carlsbad, CA); F_x_Cycle Propidium Iodide/RNase (PI) solution from Life Technology (Grand Island, NY); Cell tracker, orange-fluorescent CMTMR (CFDA-SE), 5-(and-6)-Carboxyfluorescein Diacetate, Succinimidyl Ester (CFDA SE) green tracking dye, 7-Aminoactinomycin D (7AAD), Pyronin Y, DAPI (4',6-Diamidino-2-Phenylindole, Dihydrochloride), CellTrace Far Red tracking dye and Texas Red-X phalloidin from Molecular Probes (Eugene, OR).

**Antibodies and Cytokines**

All antibodies were raised against human proteins. Rabbit anti-E-cadherin, -Connexin (Cx)43, -vimentin, -α/β-tubulin, –GAPDH and –phospho p65 were purchased from Cell Signaling Technology (Boston, MA); anti-TLR3 and -TLR4 from United States Biochemical (Swampscott, MA), human prolyl 4 hydroxylase (5B5) from DAKO North America (Dako, Denmark), phycoerythrin (PE)-conjugated murine anti-human CD44 from BD Bioscience (San Jose, CA), PE-rabbit anti-CD86, Allophycocyanin (APC)- APC-murine anti-CD90, secondary goat/rabbit APC-anti murine IgG1, anti-PerCP (Peridinin-Chlorophyll) CD31, FITC- murine anti-CD86, APC- murine anti-CD11b, APC murine anti-HLA-DR, unconjugated murine anti-HLA-DR/DP/DQ, PE murine anti-CD206, unconjugated anti-rabbit CD206, APC murine anti-CD209, PE-human-anti-CD9, FITC-human-anti-CD63-FITC, APC-human-anti-ALIX, isotypes conjugated to FITC, APC and PE from BD Pharmingen (San Jose, CA), anti-rabbit Vinculin from Abcam (Cambridge, MA), HRP-anti-GFP and murine anti-Cx-32/-26 from Zymed (San Francisco, CA), Texas Red donkey anti-mouse IgG from Santa Cruz (Santa Cruz Biotechnology, Dallas Texas) and Alexa Fluor 405 anti-rabbit IgG from Life Technologies. Recombinant human M-CSF, IFNγ and IL-4 were purchased from R & D systems (Minneapolis MN).

**Cell Lines**

MDA-MB-231 and T47D BCCs were purchased from American Type Culture Collection (ATCC) and cultured as per ATCC instructions. All cell lines used in this study were tested by Genetica DNA Laboratories (Burlington, NC). Both cells were validated as the original cells using ATCC STR database (www.atcc.org.org/STR _Database.aspx)). HBL-100 was provided by Dr. Chen Liu, Department of Pathology and Laboratory Medicine, Rutgers New Jersey Medical School.

# Sorting of BCC subsets

BCCs were stably transfected with pEGFP1-Oct3/4 and then selected based on GFP intensity, as described ^1^. The cells with relative GFP intensities were sorted with the FACSAria II cell sorter (BD Biosciences). The sorted populations were designated Oct4^hi^, Oct4^med^ and Oct4^lo^.

**Culture of MSCs**

### MSCs were cultured from human BM aspirates, as described ^2^. Briefly, unfractionated aspirates were diluted in DMEM containing 10% FCS and then added to vacuum gas plasma-treated plates (BD Falcon; Franklin Lakes, NJ). After 3 days, red blood cells and granulocytes were removed by Ficoll-Hypaque density gradient centrifugation. The mononuclear fraction recovered from each plate was replaced in fresh medium. At weekly intervals, 50% of the media was replaced with fresh media. The adherent cells were serially passaged five times after the growth attained ~80% confluence. After four cell passages, the adherent cells were symmetric, were CD14–, CD73+, CD44+, CD34–, CD45–, CD90+, prolyl-4-hydroxylase–, and showed multi-lineage differentiation for adipocyte and osteogenic cells.

**Gap Junction Intercellular Communication (GJIC)**

BCCs stably transfected with pGFP-Oct4A were labeled with 2.5 µM of CFDA dye (green), and the MΦs with 2.5 µM CMTMR dye (red). The BCCs and MΦs (M2a and M1) were co-cultured for 48 h at 1:1 ratio. The expression of GFP allowed us to gate the different BCC populations to analyze dye transfer. We de-adhered the cells with 0.2% collagenase and then assessed dye transfer by flow cytometry, as described ^3^.

**Western Blot Analysis**

Proteins were electrophoresed on Novex® NuPAGE® 14 % Tris-glycine gel with SeeBluePlus2 Prestained Standard (both from Invitrogen). The proteins were transferred onto nitrocellulose membrane (Invitrogen), using the iBlot® 2 Dry Blotting System (Invitrogen). The membranes were developed with anti-Cx43 and anti-MΦ markers, all at 1/1000 final dilution at 4^0^ C overnight. All secondary antibodies were HRP conjugated and were used at 1/2000 final dilution for 2 h incubation at room temperature. Chemiluminescent detection was performed using Novex® ECL Chemiluminescent Substrate Reagent Kit.

**BCC Proliferation**

Two methods were used to analyze BCC proliferation in co-cultures with MΦs, Cyquant cell proliferation and cell cycle analysis by PI staining. The Cyquant assay was performed according to manufacturer’s protocol (Molecular Probe). In this assay, the background fluorescence of cultures with MΦs alone was subtracted from the cultures with co-cultures.

**Cytokine Analyses**

Cytokines were quantitated with the V-Plex human proinflammatory panel 1 assay kit, purchased from Mesoscale Discovery (Rockville, MD) following manufacturer’s instructions.

**Scratch Assay**

BCC migration assay was performed in 60 mm tissue culture grade dishes. At 85-90% cell confluence, 1 μm scratches were made with a 20 μL pipette tip. The cells were washed with 1x PBS to remove cellular debris, followed by the addition of exosome-free media with or without exosomes from M1 or M2a MΦs. Cell migration was studied for up to 48 h or more (depending on the cell type) using EVOS FL Auto 2 Imaging System (ThermoFisher Scientific).

**Cell Cycle Analyses**

Cell cycle analyses were performed as described ^4^. Briefly, the cells were labeled with PI/RNase staining for 30 min, following manufacturer’s instructions. The cells were then analyzed by flow cytometry with the FACScalibur. Data were analyzed using ModFit software (Verity Software House). G_0_ and G_1_ phases were discerned by co-labeling with 1 µg/mL 7-AAD and 1 µg/mL Pyronin Y. The low DNA content was gated based on 7-AAD, which was further analyzed for low RNA depending on pyronin Y uptake.

# Tumorsphere assay

BCCs were seeded at one cell per well in serum-free media in 96-well low-adhesion plates (Costar, Corning, NY). At day 10, wells with spheres containing >20 cells were designated as tumorsphere+, as described ^1^.

**Immunohistochemistry**

Heat-induced antigen retrieval was performed on deparaffinized sectioned tissue for 1.5 h followed by cool down phase for 30 min. The slides were washed 3x with 1x PBS followed by incubation with 0.2% Triton X for 10 min. The slides were washed 3x in 1x PBS and blocked with 3% BSA for 30 min at room temperature in a humidified chamber. After 16 h, the slides were incubated to identify the specific cells, using antibodies to the following: GFP for Oct4 ^hi^ CSCs, anti-MHC-II for M1 MΦ and anti-MRC-1/CD206 for M2a MΦ. All antibodies were used at a final dilution of 1/1000. After 24 h of incubation, the slides were washed 3x with 1x PBS followed by incubation with secondary antibodies for 1 h at room temperature in a humidified chamber. Finally, the slides were incubated with 1/300 dilution of stock solution (0.6 mM) DAPI for 15 min, followed by washing 3x with 1x PBS. Coverslips were added for protection and samples were immediately imaged with the EVOS FL Auto 2 Imaging System.

The studies for baseline MФ type in the femurs of nude mice were conducted as above except for indirect labeling for CD206 with Alexa Fluor 405 anti-IgG both at 1/200 final dilution. The presence of M1 MФs was similar studied with unconjugated anti-MKC-II and secondary anti-mouse Texas Red IgG, both at 1/200 final dilution.

The method for non-fluorescence detection was similar except for the use of alkaline phosphatase conjugated secondary antibody, which was detected with BCIP/NBT substrate (Kirkegaard and Perry Labs, Gaithersurg, MD). The slides were counterstained with Harris Modified Hematoxylin.


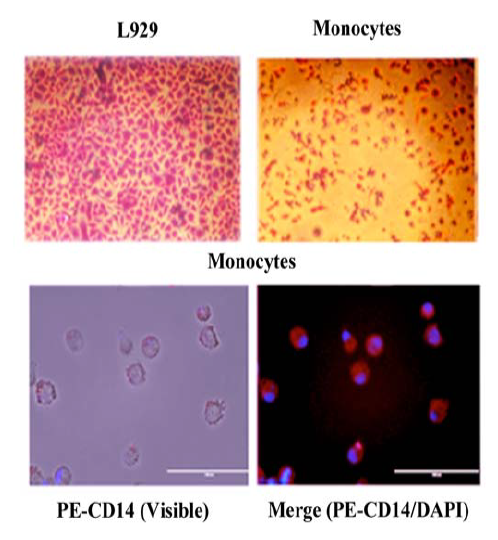


**Figure S1. Characterization of cultured monocytes.** Bone marrow mononuclear cells were isolated by Ficoll Hypaque gradient centrifugation. The monocyte fraction was isolated by anti-CD14 coupled Dynabeads. The cells were characterized by non-specific esterase (top/right panel). Positive control used L929 cells (top/left panel). Additional characterization was performed by phenotyping - immunofluorescence for CD14 (lower panels). The PE-CD14+ cells were merged with images taken with the visible light (lower/left panel). The lower right panel showed the merged images of CD14 (PE) with DAPI.


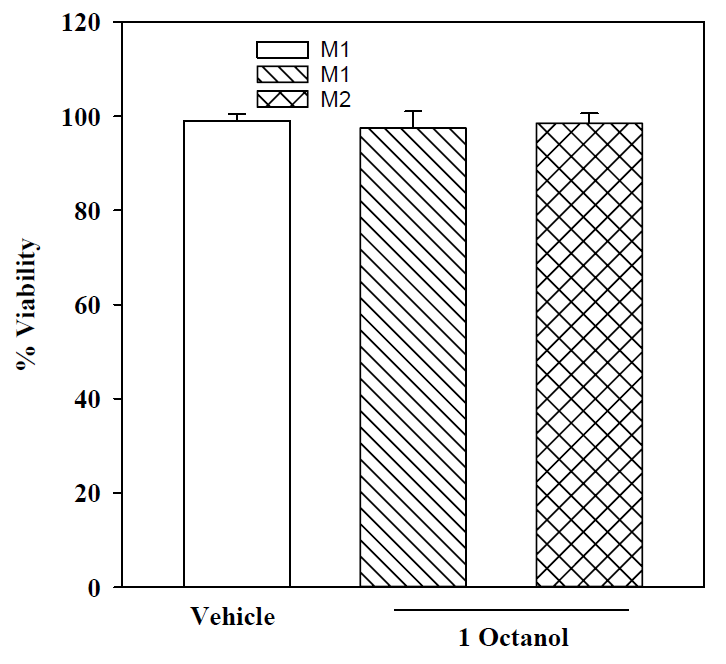


**Figure S2. Cell viability of CSCs and MΦs in the presence of 1-Octanol.** Co-cultures of M1 or M2 MΦs were established at 1:1 ratio with CSCs from MDA-MB-231. After 72, cell viability was assessed using cell titer blue. The results show the mean % viable cells±SD, n=3.

**M2 MΦ**

**M1 MΦ**


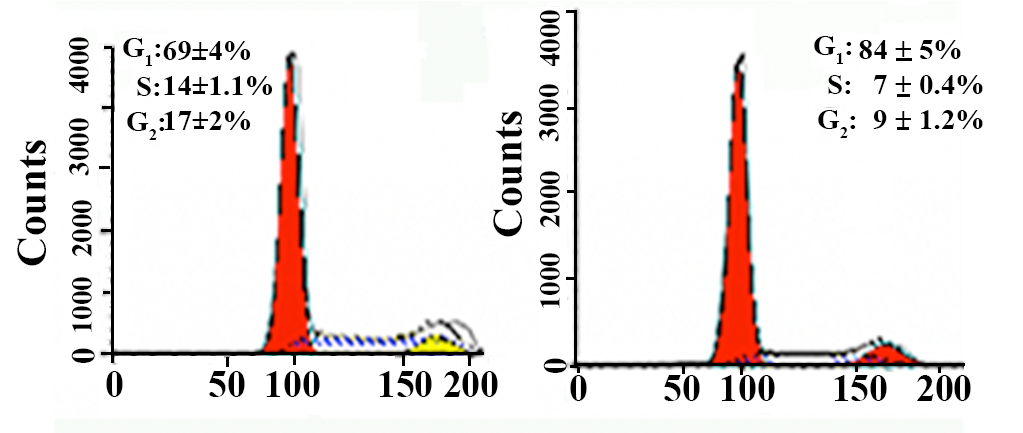
**Figure S3:** **Effects of MΦ phenotypes on the cycling of CSCs in co-cultures with stroma.** M1 or M2 MΦs were added to co-cultures of CSCs from MDA-MB-231 and BM stroma at 1:1 ratio. After 72 h, cell cycle analyses were done by propidium iodide. The CSCs were analyzed by gating on cells labeled for pan-cytokeratin. The figure represents three independent experiments for MDA-MB-231 and T47D. The percentages of cells in the different cycling phases are the mean±SD.

**
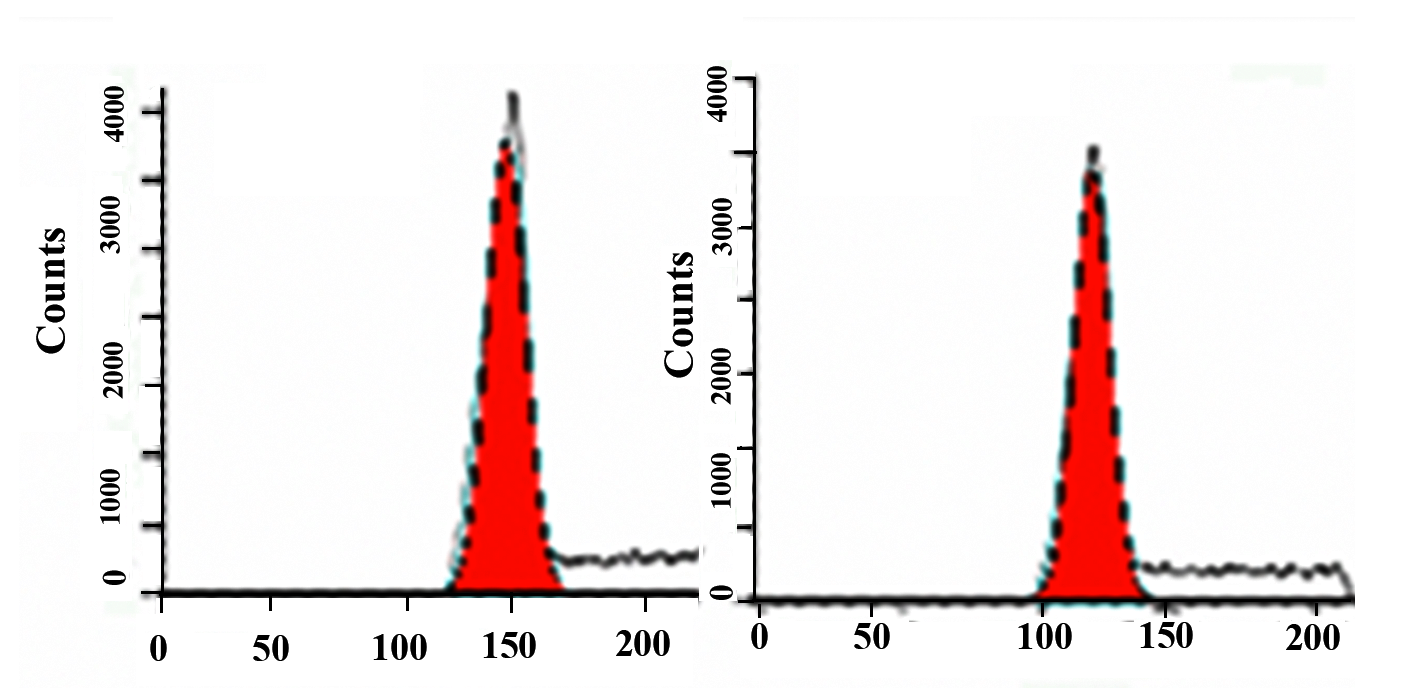
**

**M2 MΦ**

**M1 MΦ**

**Figure S4.** **Effects of MΦ types on the cycling of BM stromal fibrobasts.** M1 or M2 MΦ were added to co-cultures of CSCs and BM stroma. After 72 h, cell cycle analyses were done by propidium iodide. The fibroblasts, which comprised of the major cell subsets were analyzed by gating on cells labeled for pan-PH4. The figure represents three independent experiments for MDA-MB-231. The percentages of cells in the different cycling phases are the mean±SD.


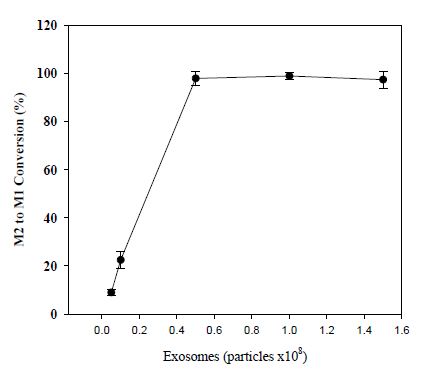


**Figure S5. Dose-response curve of exosome concentrations versus M2 MΦ conversion to M1 type.** Different concentrations of exosomes from LPS-stimulated MSCs were added to M2a MΦs. After 72 h, the percentages of M2a MΦs expressing MHC-II (M1 MΦs) were calculated. The results are the mean±SD of three independent experiments. The MΦs were established from bone marrow mononuclear cells.

**References**

1. Patel, S. A. *et al.* Delineation of breast cancer cell hierarchy identifies the subset responsible for dormancy. *Scientific Rep* **2**, 906 (2012).
2. Kapoor, S. *et al.* Tolerance-like mediated suppression by mesenchymal stem cells in patients with dust mite allergy–induced asthma. *J Allergy Clin Immunol* **129**, 1094-1101 (2012).
3. Park, J. M. *et al.* Exogenous CXCL12 activates protein kinase C to phosphorylate connexin 43 for gap junctional intercellular communication among confluent breast cancer cells. *Cancer Lett* **331**, 84-91 (2013).
4. Lim, P. K. *et al.* Gap Junction–Mediated Import of MicroRNA from Bone Marrow Stromal Cells Can Elicit Cell Cycle Quiescence in Breast Cancer Cells. *Cancer Res* **71**, 1550-1560 (2011).
